# Separation-related behavior of dogs shows association with their reactions to everyday situations that may elicit frustration or fear

**DOI:** 10.1038/s41598-021-98526-3

**Published:** 2021-09-28

**Authors:** Rita Lenkei, Tamás Faragó, Viktória Bakos, Péter Pongrácz

**Affiliations:** Department of Ethology, ELTE Eötvös Loránd University, Budapest, Hungary

**Keywords:** Zoology, Animal behaviour

## Abstract

Separation related disorder in dogs is a multi-faceted phenomenon. Dogs can react to the absence of their owner due to different inner states such as fear, panic or frustration. We hypothesized that dogs that are prone to frustration or fearfulness in other contexts would show a different behavioral response to separation from the owner. We investigated the association between inner states in different contexts and separation behaviors by combining a questionnaire with a separation test. Fear-related questionnaire components were rather associated with whining and the absence of barking. Dogs that received higher scores in the demanding component of the questionnaire, which might be in association of the frustration threshold of the dog, barked more and were more likely to scratch the door. Finally, dogs that were more prone to phobic reactions whined somewhat more and tried to escape. We provide empirical support for the assumption that separation-related behavioral responses of dogs might be triggered by different emotions.

## Introduction

One of the main eliciting factors of the manifestation of canine attachment behavior complex, is that the dog experiences a moderate level of stress in the absence of the owner^1^. Normally, this mild level of stress should not cause welfare problems in pet dogs, however in some cases the stress reaction is so intense that it can be considered as a behavior problem^2^. Separation related problems (SRP) are among the most common behavior disorders in family dogs, not only affecting the dog itself, but they can also be disturbing to the owner and the environment^2,3^. Although there are approaches to alleviate symptoms in affected dogs, such as medications and behavioral therapy (e.g.:^4–7^), these treatments are often long and demanding, and in some serious cases inefficient, which sometimes leads to the relinquishment of the dog^6^. Consequently, SRP represents a major welfare problem and its investigation is important both from ethological and veterinary perspectives.

There is abundant literature about the possible causes and risk factors of SRP (for a review see:^8^). Both genetic^9,10^ and environmental factors affect separation-related behavior, such as the attitude of the owner towards the dog^11,12^ or earlier negative experiences^13^. The association between SRP and demographic factors such as sex or neutered status of the dog^14,15^ was also suggested. The stress-related symptoms can be highly variable—up to 54 signs were identified in a questionnaire study^16^. Some of these behaviors are specific to the problem, such as the destruction of objects, escape attempts, or intensive vocalization such as whining, barking or howling. There are more general stress indicators as well, such as salivation or inappropriate urination and defecation. Although the signs and possible causes are well described, there remains many open questions as the appearance and relative strength of the signs often varies between individuals. For instance, it is known that some affected dogs constantly pace when left alone while others rather show inactivity^17^. These contradictions of the different signs, the multicausality, and the presence of several different risk factors, illustrate the complexity of this phenomenon, suggesting that it cannot be handled as one demarcated behavior problem. Instead, SRP is rather an umbrella term for any stress related behaviors when the dog is left alone by the owner.

Based on the wide array of various stress signs, one can hypothesize that separation stress could be associated with several inner states in the dog, and the relative intensity of these inner states can modify the actual behavioral phenotype seen during the separation episode. Firstly, it became established that not only anxiety could be responsible for the separation behavior. This new approach is strengthened by the theory that individual differences might affect either how one perceives the particular situation or the type of emotion it elicits^18^. This realization eventually resulted in the change of the previously used term ‘separation-anxiety’, to more general terms such as ‘separation related disorder’ or ‘separation related problem’. For example, Sherman and Mills^19^ state that “The term separation distress may best describe the phenomenon, which incorporates signs consistent with anxiety, fear, and phobic behavior”. While these terms are often used interchangeably in the literature, the underlying neural mechanisms and their behavioral manifestations may be different^19^. Anxiety is a reaction to an upcoming threat or uncertainty that causes behavioral and physiological signs of stress. Besides anxiety, fear can also elicit stress during separation, as it is an emotional reaction to a direct threat or stimuli generating defensive behaviors or avoidance. Finally, phobia is a persistent intensive fear reaction evoked by well described situations or objects^19–21^. There are several indications that fearfulness, as a personality trait, has a connection with SRP. Based on questionnaire studies, generally fearful dogs had a higher risk of developing SRP, furthermore noise-phobia and thunderstorm-phobia are also thought to be connected to it^19,21,22^.

In addition to the fear-related inner states, frustration is another likely candidate for the emotional background of separation-related behaviors in dogs. Frustration is a negative affective state that appears when an animal’s expectation is violated, for example, when a needed resource is inaccessible, or when the reward of a previously reinforced behavior ceases. Frustration manifests itself in several contexts and may have an important role in behavior problems^23^. If we consider the owner as one of the most important resources for the dog, it is reasonable to assume that they might experience frustration during separation (i.e. when the access to the owner is denied). The fact that food-related aggression is connected to the appearance of SRP strengthens the theory that a more intense resource holding motivation is somehow associated with SRP^15^.

Lund and Jørgensen^24^ developed a theoretical model about separation stress. According to this, separation triggers frustration that on one hand elevates arousal, resulting in excessive barking and increased explorative behaviors, which in turn may become disorganized, resulting in the destruction of objects^25^. Meanwhile, frustration and the individual features of the dog such as prior experiences, trigger fear that causes other signs of separation stress such as salivation, whining or escape attempts. While they found several correlations between particular signs of SRP, they could not make any clear distinction between different groups of dogs with SRP. However, recently de Assis et al.^16^ in an extensive and complex questionnaire study involving more than 2700 dogs, determined seven factors from the different symptoms and identified four main sub-populations of dogs with SRP. Their conclusion was based on combinations of factors, such as exit frustration (destructive behaviors towards the doors or windows), reactive communication, or signs of social panic. Although their results are compelling and shed light on the complexity of the phenomenon, they admit that it is not applicable to describe the causal relationships and the underlying motivational background of the signs that they have identified.

Despite the growing interest and importance of this question from the aspect of applied approaches, there is still no direct evidence that different inner states indeed cause different stress signs during separation. As a potentially promising proxy, it was found in previous studies that dogs’ vocalizations may help with identification of specific inner state involvement in the background of SRP^26^. According to this, frustration may elicit mostly barking, while whining would be more indicative of fear^12,24,27^. It is assumed that the owner’s inconsistency (i.e. unpredictable responses) during his/her interactions with the dog could cause a reduced frustration threshold in the dog as we found that in a separation test, dogs that barked frequently but did not whine, had the most lenient owners^12^. In another study we found that cooperative breeds, which work in close visual contact with their handler^28^, barked more frequently during separation than the independently working dog breeds^29^. Although there is no difference in the two breed groups’ attachment patterns^30^, this possibly suggests that functional breed selection may not only have resulted in dogs that are more motivated to stay close to their owners, but could also make them more prone to frustration in the case of being separated from their owner^29^. On the other hand, dogs with owner-reported SRP started to whine sooner and more likely during separation than the non-SRP dogs, supporting the theory that anxiety and fear is indeed associated more often with whining than with barking^27^.

In this study we aimed to determine the possible connection between different inner states and the dog’s behavior in the absence of the owner. We hypothesize that different inner states manifest themselves in different signs of stress during the separation. As affective states in a particular context are influenced by the individual’s a priori experiences^18^ we predicted that dogs that show fearfulness in other everyday situations will behave differently during separation from their owners, than dogs that seem to have a low tolerance of frustration. We developed an online questionnaire to assess the general fearfulness and threshold of frustration tolerance of the dogs. In addition, we tested dogs in an indoor separation situation (developed by Konok et al.^31^) to describe their separation behavior. We performed two separate Principal Component Analyses (PCA) on the questionnaire items and the behavioral variables from the separation test. Finally, we were looking for associations between the fearfulness or frustration scores and the dogs’ behavior in the separation test. We also looked for possible associations between the first occurrence of whining, barking, scratching the door, rearing at the door and the questionnaire scores.

## Results

### Results of the PCA analysis of the questionnaire

Out of 59 questions, the analysis resulted in 7 main components and based on Cronbach’s alpha values each turned out to be reliable. The components together explained 56.3% of the total variance. For further reference and for easier discussion we labelled the components with fitting names based on the consisting components (see Table 1). The first factor (‘Relaxed’, Cronbach’s alpha: 0.91) contained variables mostly associated with stress-tolerance in new environments or situations. The second factor (‘Obedient’, 0.89) consisted of twelve items, all related to obedience and tractability. The six items of the third trait (‘Fear dogs’, 0.91) were associated with fearful behaviors in the presence of unfamiliar dogs. The fourth component (‘Fear humans’, 0.92) with its six items was mostly related to fearfulness with unfamiliar humans. The fifth component (‘Demanding’, 0.77) consisted of variables associated to various demanding behaviors of the dog, either food-related ones, or getting involved in some joint activity with the owner. The sixth (‘Protest dislike’, 0.77) factor was associated with stubborn behaviors of the dog. While the last, seventh component (‘Have phobias’, 0.78) consisted of items related to phobic behaviors, including noise and thunderstorm phobias.

**Table 1:** The results of the principal component analysis of the questionnaire (N = 392). In case of each principal component, we highlighted the loading of those items with bold that significantly contributed to that component.

Questionnaire item	Relaxed	Obedient	Fear dogs	Fear humans	Demanding	Protest dislike	Have phobias	Origin
Does your dog tolerate being held down	**0.507**	− 0.046	0.058	0.095	− 0.073	− 0.284	− 0.008	
Dog is emotionally stable, not easily upset (neuroticism/emotional reactivity)	**0.686**	− 0.019	− 0.139	− 0.046	0.109	− 0.162	− 0.085	Temesi et al
Dog remains calm in tense situations (neuroticism/emotional reactivity)	**0.679**	0.134	− 0.193	0.028	0.054	− 0.052	− 0.09	Temesi et al
Dog gets nervous easily (neuroticism/emotional reactivity)	− **0.6**	0.035	0.221	− 0.091	− 0.03	0.277	0.125	Temesi et al
Dog can be tense (neuroticism/emotional reactivity)	− **0.654**	0.062	0.112	0.003	− 0.032	0.196	0.109	Temesi et al
Dog is relaxed, handles stress well (neuroticism/emotional reactivity)	**0.657**	− 0.011	− 0.21	0.003	− 0.019	0.054	− 0.183	Temesi et al
Your dog usually appears relaxed (negative activation)	**0.767**	0.005	− 0.008	0.026	− 0.053	− 0.082	0.119	Temesi et al
Your dog appears calm in unfamiliar environments (negative activation)	**0.805**	0.114	0.013	− 0.093	− 0.011	0.066	0.153	Temesi et al
Your dog appears calm in noisy, crowded places (negative activation)	**0.754**	0.152	0.115	− 0.133	0.069	0.132	− 0.126	Temesi et al
Dog is anxious (fearfulness)	− **0.677**	− 0.003	0.018	0.176	0.104	− 0.056	0.186	Temesi et al
Dog adapts easily to new situations and environments (fearfulness)	**0.632**	0.066	− 0.005	− 0.302	− 0.029	0.127	0.008	Temesi et al
I can enforce my will on my dog	0.109	**0.652**	− 0.087	0.133	− 0.026	− 0.039	− 0.077	Lenkei et al
The owner can easily stop unwanted activities (e.g. by verbal inhibition)	0.068	**0.757**	0.003	0.036	− 0.007	− 0.075	0.149	Bálint et al
Obeys commands for the first time	− 0.017	**0.765**	− 0.124	0.028	− 0.008	− 0.006	− 0.089	
The dog can be called back even if there are other dogs in its vicinity	− 0.041	**0.842**	0.013	− 0.097	− 0.041	0.056	− 0.01	Bálint et al
The dog can be called back even if there are other dogs, animals (e.g.: pigeon, cat) in its vicinity	0.004	**0.791**	0.109	− 0.044	0.03	− 0.012	0.02	Bálint et al
Once the dog understands that something is forbidden, it is easy to prevent the same thing on a subsequent occasion	0.245	**0.606**	0.036	0.043	− 0.125	0.043	0.09	Bálint et al
Does not, or almost never obeys commands	0.004	− **0.688**	0.166	− 0.066	0.008	0.101	0.004	Lenkei et al
The dog can be called back even if there are humans in its vicinity	− 0.09	**0.778**	0.056	− 0.033	0.017	0.021	− 0.084	Bálint et al
It is hard to lead away the dog on leash if its attention is focused on food, other dogs or humans	− 0.076	− **0.544**	− 0.002	− 0.018	0.182	0.061	− 0.012	
Sometimes the dog’s attention is so distracted that it impairs its obedience	− 0.076	− **0.536**	0.086	− 0.065	− 0.01	0.145	0.014	Bálint et al
The dog dislikes (pulls on the lead, jumps, barks or whines) when it is not allowed to approach another dog, human or object	− 0.157	− **0.414**	0	0.006	0.028	0.236	− 0.119	
Dog takes it badly when something is forbidden	− 0.11	− **0.442**	0.045	0.109	0.169	0.408	− 0.083	
Dog acts anxiously or fearfully when approached directly by an unfamiliar dog of the same or larger size (social fear)	− 0.059	0.004	**0.889**	− 0.009	− 0.005	− 0.019	− 0.01	Temesi et al
Dog behaves fearfully towards other dogs (fearfulness)	− 0.004	− 0.018	**0.955**	0.035	− 0.016	0.009	− 0.039	Temesi et al
Dog acts anxiously or fearfully when barked, growled or lunged at by an unfamiliar dog (social fear)	0.036	− 0.004	**0.92**	− 0.049	0.02	− 0.087	0.052	Temesi et al
Dog acts anxiously or fearfully when approached directly by an unfamiliar dog of smaller size (social fear)	0.046	− 0.068	**0.883**	0.079	− 0.007	0	− 0.018	Temesi et al
Dog acts anxiously or fearfully when unfamiliar dogs visit your home (social fear)	− 0.053	0.022	**0.821**	0.164	− 0.013	0.01	− 0.012	Temesi et al
Dog avoids other dogs (fearfulness)	− 0.042	0.057	**0.646**	0.045	0.077	0.033	0.035	Temesi et al
Dog acts anxiously or fearfully when an unfamiliar person tries to touch or pet the dog (social fear)	− 0.048	0.028	0.03	**0.91**	− 0.026	0.034	− 0.009	Temesi et al
Dog acts anxiously or fearfully when approached directly by an unfamiliar adult while away from home (social fear)	− 0.032	− 0.019	0.053	**0.93**	− 0.015	0.007	0.011	Temesi et al
Dog behaves fearfully towards unfamiliar people (fearfulness)	0.03	− 0.022	0.027	**0.958**	− 0.005	0.002	− 0.002	Temesi et al
Dog acts anxiously or fearfully when unfamiliar persons visit your home (social fear)	− 0.02	− 0.052	0.044	**0.907**	− 0.029	0.035	0.059	Temesi et al
Dog acts anxiously or fearfully when approached directly by an unfamiliar child while away from home (social fear)	− 0.004	0.101	0.034	**0.877**	0.099	0.027	− 0.015	Temesi et al
The dog has a skill to seek out and steal food from anywhere, sometimes even from the hands of people	− 0.044	− 0.09	− 0.106	− 0.072	**0.562**	0.136	− 0.039	Bálint et al
If there is food on the table, does your dog beg for it	0.04	− 0.041	0.026	− 0.022	**0.862**	− 0.029	0.012	Lenkei et al
Does your dog get excited before his/her regular feeding time	− 0.145	0.163	0.071	− 0.029	**0.678**	0.064	− 0.09	Lenkei et al
Is your dog begging for food while you are eating	0.084	− 0.14	− 0.014	0.04	**0.805**	− 0.101	0.012	Lenkei et al
Is your dog begging for treats/food if it knows the place where these are kept	0.042	0.06	− 0.073	0.075	**0.748**	0.007	0.082	Lenkei et al
The dog becomes overly excited, when it anticipates an upcoming pleasant activity (walking, feeding, playing)	0.006	− 0.02	0.024	0.037	**0.345**	0.271	0.017	
Does your dog get excited when he/she is left confined alone in another room/house? (while you are still around, just not with your dog)	− 0.06	− 0.088	0.083	− 0.039	**0.462**	0.246	0.04	Lenkei et al
The dog shows its intention to enter a room, when the door is closed	0.045	− 0.105	0.139	− 0.018	**0.425**	0.239	0.086	
Does your dog bring a toy for you when he/she wants to play	0.131	0.052	0.133	− 0.084	**0.352**	− 0.006	0.08	Lenkei et al
The dog responds by barking or growling to situations/events it does not appreciate or opposes	0.038	− 0.009	− 0.088	0.285	0.065	**0.672**	0.014	Bálint et al
The dog responds threateningly/shows intimidating behavior when being punished or disciplined	0.095	− 0.11	− 0.089	0.082	− 0.048	**0.754**	0.086	Bálint et al
If the dog wants to obtain something, it pursues that persistently or even aggressively	− 0.051	− 0.014	0.023	0.008	0.174	**0.661**	− 0.092	Bálint et al
Does your dog snap at your hand or leg when you discipline or punish it	− 0.068	− 0.192	0.049	− 0.027	− 0.003	**0.697**	0.171	
Dog can be moody	− 0.209	0.092	0.068	0.03	0.137	**0.459**	− 0.267	
The dog does not tolerate being physically restrained	− 0.397	0.061	0.023	− 0.053	0.07	**0.482**	0.066	
The dog often barks in unusual or novel situations. In these cases, it is almost impossible to calm it	− 0.056	− 0.222	0.084	0.107	− 0.133	**0.428**	− 0.02	Bálint et al
Your dog appears nervous and/or jumpy for several minutes after it has been startled (negative activation)	− 0.281	− 0.019	− 0.029	0.114	0.154	− 0.08	**0.577**	Temesi et al
Dog acts anxious or fearful in response to sudden or loud noises (non-social fear)	− 0.242	0.023	0.003	0.101	0.004	− 0.01	**0.734**	Temesi et al
Dog acts anxious or fearful during thunderstorms (non-social fear)	0.071	− 0.033	− 0.065	− 0.074	0.007	0.023	**0.917**	Temesi et al
Your dog has a specific fear or phobia (negative activation)	0.032	0.053	0.138	0.103	− 0.012	0.097	**0.762**	Temesi et al
Cronbach’s alpha	0.91	0.89	0.91	0.92	0.77	0.77	0.78	
Variance explained	10.5%	10.4%	8.9%	8.6%	6.7%	6.2%	5%	

The questions related to fearfulness of dogs are based on a consensus questionnaire of 6 different questionnaires, for the origin of particular items see Temesi et al.^45^.

### Results of the PCA analysis of the behavioral variables from the separation test

As a result of the analysis of the behavior test five main components emerged (see Table 2). The components together explained 56.3% of the total variance. All were reliable when based on Cronbach’s alpha values. The first component (‘Chair’, Cronbach’s alpha: 0.7) consisted of four variables associated to the owner’s chair in the room. The second component (‘Escape’, 0.64) contained four items most of which are escape-related behaviors such as rearing to the door or moving. The variables in the third component (‘Whining–door’, 0.57) are whining and exploration of the door and the absence of laying down. The fourth component (‘Bark–Wagging’, 0.52) consists of five items including barking and tail wagging. While the last, fifth component (‘Sit’, 0.69) consists of sitting and also standing as a negatively scored item.

**Table 2:** The results of the Principal Component Analysis of the separation test (N = 167; based on Marx et al.^26^). In case of each principal component, we highlighted the loading of those items with bold that significantly contributed to that component.

Behavior	Chair	Escape	Whine–door	Bark–wagging	Sit
Door distance	− **0.742**	0.159	0.123	− 0.123	0.070
Chair exploration	**0.708**	0.048	0.174	− 0.167	0.002
Chair distance	**0.694**	− 0.243	− 0.139	0.361	0.014
Chair orientation	**0.659**	0.141	0.091	− 0.276	0.179
Rearing	− 0.106	**0.832**	− 0.093	0.115	0.053
Scratching	− 0.204	**0.720**	0.115	0.134	0.081
Panting	0.304	**0.523**	− 0.102	− 0.220	− 0.036
Moving	0.302	**0.494**	0.387	− 0.219	− 0.169
Laying	− 0.052	− 0.065	− **0.705**	− 0.022	0.209
Whining	0.025	− 0.145	**0.654**	0.287	0.246
Door exploration	− 0.197	0.063	**0.635**	− 0.189	− 0.041
Barking/yelping	− 0.033	0.470	− 0.014	**0.610**	− 0.059
Tail-wagging	0.203	0.334	0.220	**0.590**	− 0.105
Exploration in general	0.086	0.059	0.289	− **0.528**	− 0.186
Door orientation	− 0.271	− 0.108	0.274	**0.471**	0.064
Other vocalization	0.064	0.168	0.148	**0.349**	− 0.031
Standing	− 0.088	− 0.195	0.285	0.084	− **0.833**
Sitting	− 0.039	− 0.109	0.242	0.049	**0.858**
Cronbach’s alpha	0.7	0.64	0.57	0.52	0.69
Variance explained	13.2%	12.7%	10.8%	10.3%	9.3%

### Results of the generalized linear models

#### ‘Whining–door’ component

We found that dogs that gained low scores in the case of this component were generally calmer (‘Relaxed’: B ± SE =  − 0.464 ± 0.13; t =  − 3.579, *p* < 0.001); and as a weak tendency ‘Whining–door’ positively correlated with other phobic behaviors (‘Have phobias’: B ± SE = 0.224 ± 0.123; t = 1.736, *p* = 0.088).

#### ‘Escape’ component

**Figure 1:**
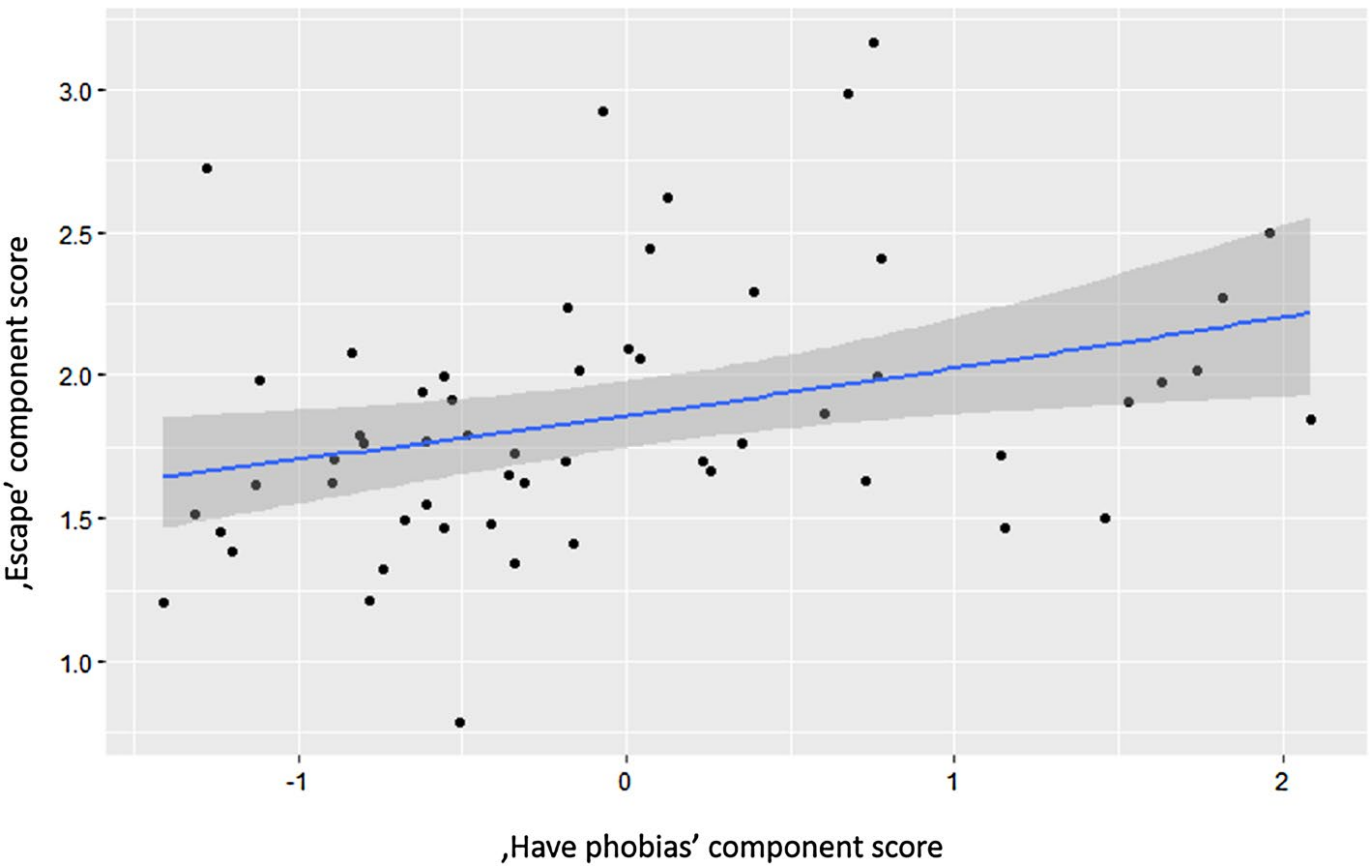
The relationship between 'Have phobias' and 'Escape' component scores.

We found that dogs with higher scores on the ‘Relaxed’ component showed somewhat less escaping behaviors (‘Relaxed’: B ± SE =  − 0.062 ± 0.034; t =  − 1.856, *p* = 0.07), while dogs with high scores of phobias presented significantly more escaping behaviors (‘Have Phobias’: B ± SE = 0.086 ± 0.032; t = 2.706, *p* = 0.009, Fig. 1).

#### ‘Chair’ component

One significant result emerged, namely that obedient dogs spent more time near it and explored more frequently the owner’s chair (‘Obedient’: B ± SE = 0.304 ± 0.126; t = 2.419, *p* = 0.019).

#### ‘Sit’ component

We also found that generally calmer dogs sit more during the separation test (‘Relaxed’: B ± SE = 0.212 ± 0.077; t = 2.772, *p* = 0.008).

#### ‘Bark–wagging’ component

**Figure 2:**
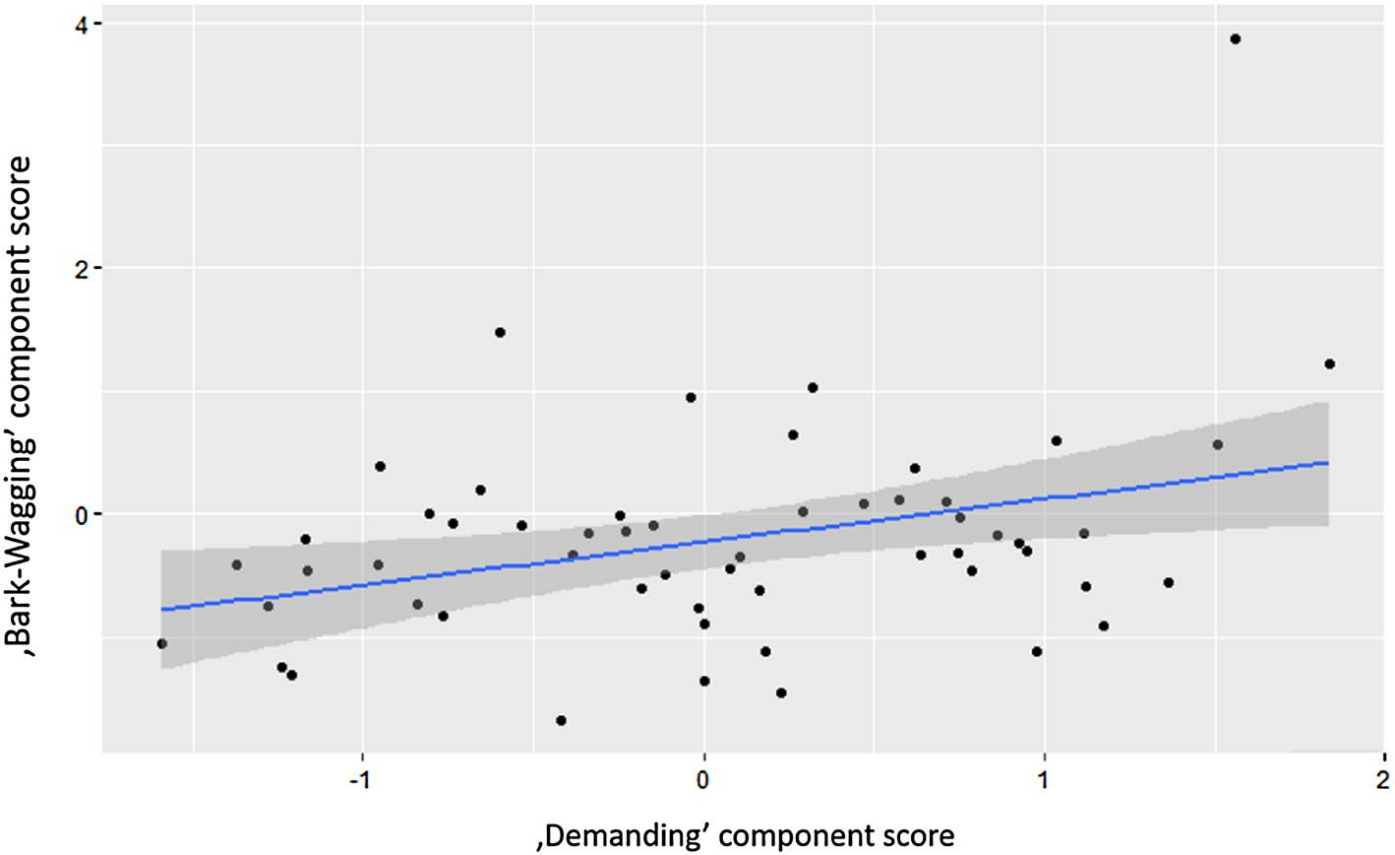
The relationship between ‘Demanding’ and 'Bark–Wagging' component score.

In the case of the ‘Bark-Wagging’ component, we found a strong positive association with frustration-related behaviors (‘Demanding’: B ± SE = 0.06 ± 0.017; t = 3.532, *p* < 0.001, Fig. 2). While dogs that fear other dogs (‘Fear of dogs’: B ± SE =  − 0.045 ± 0.017; t =  − 2.693, *p* = 0.009) and dogs that have phobias had lower ‘Bark–Wagging’ scores (‘Have phobias’: B ± SE =  − 0.070 ± 0.016; t =  − 4.468, *p* < 0.001).

### Results of the Cox-regressions

**Figure 3:**
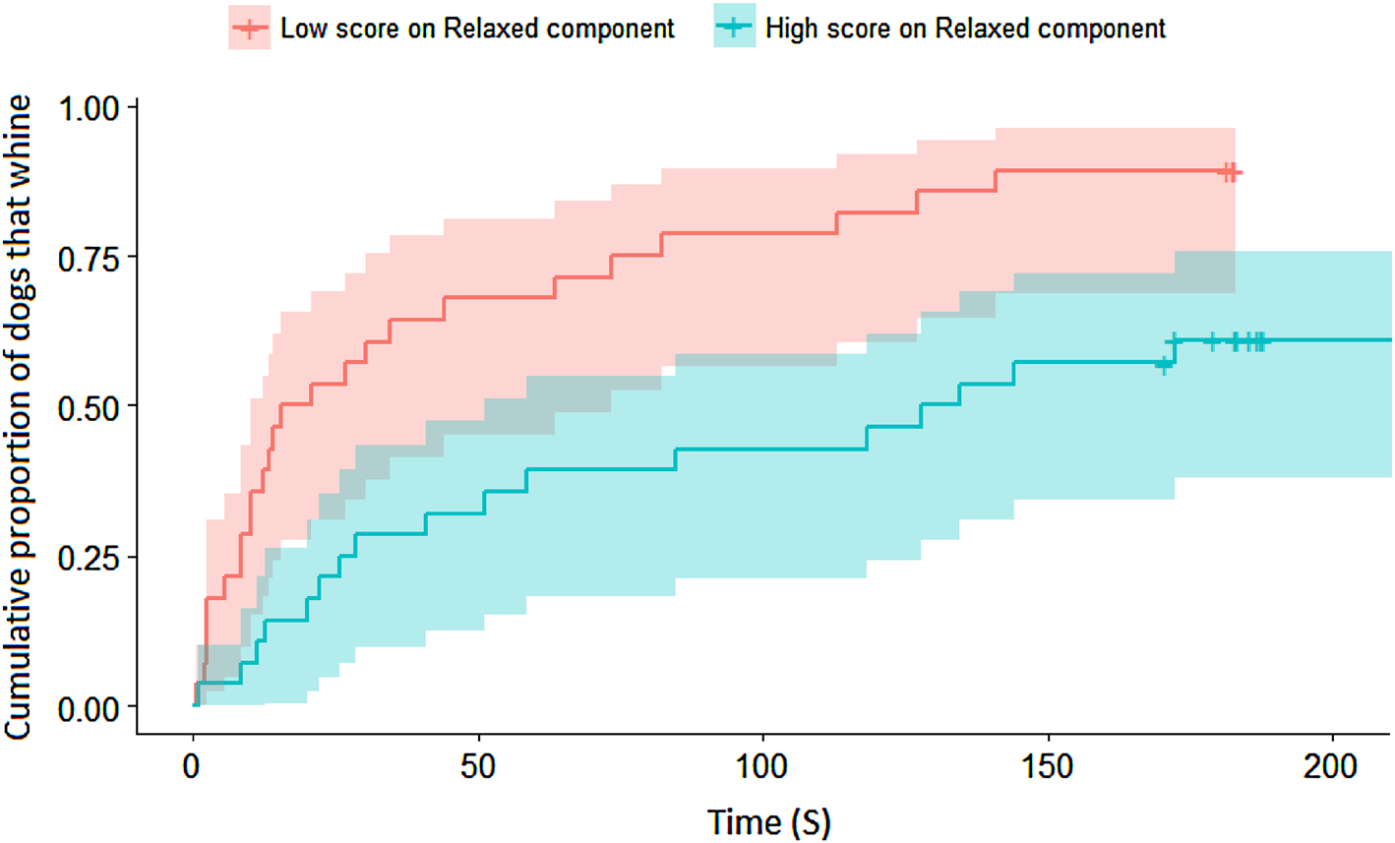
Differences based on the 'Relaxed' component in the probability of whining during the separation test.

**Figure 4:**
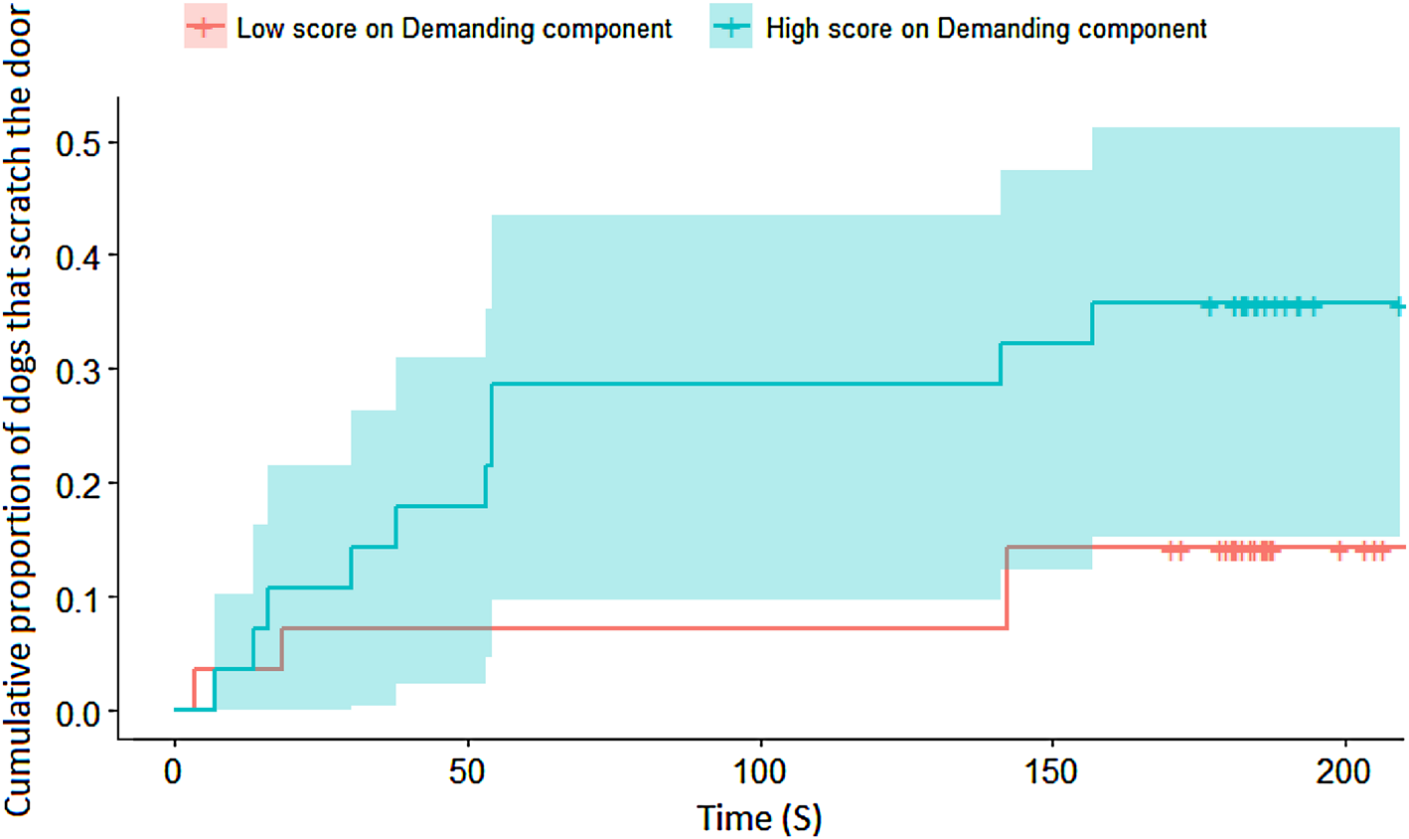
Differences based on the ‘Demanding’ component in the probability of scratching the door during the separation test.

We found that the generally calmer dogs (‘Relaxed’: exp(*β*) = 0.493 [0.35; 0.70], *z* =  − 4.031; *p* < 0.001, Fig. 3) started to whine later, while dogs that have a fear of unfamiliar humans show a tendency to whine earlier (‘Fear of humans’: exp(*β*) = 1.262 [1; 1.644], *z* = 1.728; *p* = 0.084). Additionally, we found that calmer dogs started to bark later (‘Relaxed’: exp(*β*) = 0.348 [0.165; 0.732], *z* =  − 2.780; *p* = 0.005), while dogs that show demanding behaviors in other situations started to bark earlier (‘Demanding’: *exp*(*β*) = 5.221 [1.889; 14.432], z = 3.186; *p* = 0.001). We found that obedient dogs started to scratch the door significantly later (‘Obedient’: *exp*(*β*) = 0.539 [0.301; 0.966], z =  − 2.074; *p* = 0.038); a similar, but only trend-level association was found in the case of those dogs that have fear of unfamiliar dogs (‘Fear of dogs’: *exp*(*β*) = 1.689 [0.971; 2.936], z = 1.855; *p* = 0.06). Demanding dogs started to scratch the door significantly earlier (‘Demanding’: exp(β) = 2.134 [1.078; 4.224], z = 2.174; *p* = 0.03, Fig. 4). We did not find any significant result in the case of rearing on the door.

## Discussion

Interest has been growing in the multi-faceted etiology and the possible identification of different sub-types of separation-related behavior problems in companion dogs. Our core hypothesis was that the variability of the diverse signs—at least partially—can be explained by the different underlying affective states. Though it is a relationship between two adult individuals, the human–dog social bond is thought to be analogous to filial attachment^1^. As humans provide resources to the dog—same as parents provide it to their offspring—the dog is dependent on humans and motivated to stay close to its owner. This motivation manifests itself as a stress response in the absence of the owner. However, these stress related behaviors can be various in appearance and intensity in isolation; fear, anxiety, panic and also frustration might appear. While there are several questionnaire studies about the mutual occurrence of different behavior problems—such as fearfulness or phobias—and SRP, and there are also some theoretical works about the possible inner states in the background, by our knowledge, our study is the first to combine a questionnaire with a behavior test which provides evidence of the association between different behavioral responses and presumed inner states in the absence of the owner.

Dogs that can be characterized with fearful tendencies based on their questionnaire-based scores, whined sooner and more often, but did not bark during the behavior test. We also found that dogs that scored lower on the ’Relaxed’ component showed a tendency to start to bark sooner than the more ’Relaxed’ dogs—however, the apparently relaxed behavior can be equally typical of the less fearful and less frustrated dogs as well. ‘Relaxed’ dogs also show less escape attempts such as door scratching or rearing. Contrary to this, dogs that received higher scores in the ‘Demanding’ component of the questionnaire, can be characterized with frequent and early onset barking and they also more likely and sooner scratched the door. Dogs that were told to be prone to demanding behaviors in other situations, actively and more intensively tried to get out of the room.

Items of the ‘Demanding’ component contain different contexts where the dog shows demanding behaviors like access for food, treats, playing or the company of the owner. When the expectation (including the absence or the delay of a reward) of an individual is violated frustration might appear^32^, thus it is reasonable to assume that those individuals who gained high scores on this component would also become easily frustrated if their goal is not fulfilled despite their persistence of demanding it. Therefore, frustration and persistence are strongly connected to each other as the failure of reaching the goal leads to frustration what prompts persistent efforts to pursue it^33^. Indeed, McPeake and colleagues^23^ in their extended questionnaire about frustration-related behaviors in dogs described a component called ‘Barrier frustration/Perseverance’. The fact that during their analysis questions connected to persistence and the intensive reaction when a barrier thwarted the goal of the dog formed a component is particularly interesting, as typically during separation there is a physical barrier between the dog and the owner. These results together are in line with the assumption that these dogs’ behavior might be motivated by their inability to access their owner who represents an important resource.

Based on previous questionnaire studies, it has been acknowledged that there is a co-occurrence between various noise phobias and SRP^21,34^. To our knowledge our results are the first where signs of SRP were assessed in an experimental setup and a direct relationship was found between them and various, owner-reported phobic behaviors of the dogs. Interestingly, dogs that have owner-reported phobias (‘Have phobias’) gained somewhat higher scores in case of the ‘Whining-door’ component and they also can be characterized with the absence of barking (negative association with ‘Bark–Wagging’ component), furthermore, they gained higher scores in the ‘Escape’ component. This result is in agreement with the assumption that these dogs show a different, intense panic-like reaction to separation. Unlike^21^ and^34^ Blackwell et al.^35^ found no direct association between phobias of extreme noise effects (such as thunderstorms and fireworks) and SRP, but described a connection between fear of other generic noises (that can cause startling effect in dogs) and separation-related behavioral problems. In our case, the ‘Have phobias’ component consists of items not only related to noise phobias but more generic fearful reactions of the dogs to startling stimuli. Thus, in agreement with^35^, our results provide support to the theory that dogs that are more sensitive to fear-eliciting effects would also show a specific behavioral response to separation. Here, the assumed connection with fear can be further supported with the result that these dogs tended to whine more but they seldom barked.

Affective states are usually characterized along two continuums. They are valenced either positively or negatively (valence), and they generate different levels of arousal (intensity). Not surprisingly, both frustration and fear are negative emotions, where fear and panic are thought to be higher arousal states than frustration^36^. The presumed inner states during separation are close to each other in these dimensions, particularly in the case of fear and panic where it is also known that they have a similar underlying neural mechanism^20^. While our results strengthen the theory that the cumulative experience of particular inner states also affects the reaction to separation, it is important to emphasize that naturally these mental states are not necessarily discrete, and they might overlap or change over time. Lund and Jørgensen^24^ in their model proposed the possible relation and time course of the different factors and inner states influencing separation behavior. According to their study, frustration during separation leads to barking and destructive behavior, while fear provokes general signs of distress and escape behavior. Our results partly support these, however we found that escape behaviors were rather associated with frustration and panic than with fear. It is possible that in some cases dogs show escape behaviors because they just try to leave the confined space thus their behavior response is indeed provoked by fear^37^. However, it is more likely that they rather try reestablishing the contact with their owner, thus their escape attempts might be motivated by frustration. It is also possible that Lund and Jørgensen took the frustration-related damaging of the door as ‘destructive’ behavior, whilst in our behavior test these were labelled as escape-related reactions. They also suggested that the appearance of particular behaviors during isolation are caused by the change in the dog’s emotional states (i.e. a dog at first can feel frustration, then after a while fear). Contrary to their long-term investigation in the home environment, our test lasted only 3 min and still the signs of fear and frustration could be detected in different dogs. This highlights the possibility that although dogs may experience a dynamic change in their inner state during a longer period of separation, their individual propensity for an almost immediate fear- or frustration-driven reaction can result in very different reactions to even a short interval of isolation.

As we expected, all but one of the factors that emerged from the questionnaire were somehow associated with vocalizations. Vocalization is one of the most often mentioned signs of separation stress, with a strong association between particular acoustic features of the vocalizations emitted by isolated individuals and the possible inner state of the subject^26,38,39^. In other canid species—either pups or adult individuals—whining or howling is what mostly appears instead of barking^40–42^. Barking is a vocalization type that became abundant and acoustically versatile during the evolution of the dog^27^. It is known that it carries contextual and affective information to humans and it is emitted in several different contexts^43,44^. It was found that the particular type of barks emitted in isolation (‘left alone barks’) is especially easy to recognize and it is mostly characterized by human listeners as being ‘desperate’ and ‘fearful’^44^. Based on these findings, both vocalization types have a different role during interspecific communication between dogs and humans, thus during separation they are probably emitted with an adaptive outcome by potentially changing the owner’s behavior. Barking is a long-range vocalization type, thus in this case its function might be to capture the attention of the owner and it is provoked by the unpleasant frustrating situation^39^. The whining of dogs (being a short-range vocalization) might rather elicit caring behavior from the receivers once they reappear at the scene^41^.

Contrary to other studies, here we did not compare dogs with or without predetermined SRP-status, but instead we wanted to describe their reaction to separation from the owner with behavioral scores along various scales. With this method we tried to avoid the subjectivity of the owner’s report about his/her dog’s condition^11^. It is possible that when strong separation stress truly manifests in problematic behavior at home, this type of SRP would be mostly caused by fear. Some level of frustration is a normal reaction in most dogs to separation at any location, but it is likely that being separated from the owner at an unfamiliar place would elicit also fear from the dogs. This can result in more whining (response to fear) during the tests from those dogs that usually would rather bark at home (as being mostly frustrated).

Here we used a merged questionnaire of previous studies^12,45,46^ and we also developed new questions about frustration-related behaviors of the dogs. Compared to our previous questionnaire^12^ with the inclusion of 2 new frustration related questions a combined ‘Demanding’ component emerged that included food related behaviors, while in our previous sample these questions formed a separate component. This is not surprising as inaccessible food triggers frustration-related behaviors in dogs^47^. Meanwhile, contrary to Temesi and colleagues^45^, in our sample the questions related to phobic behaviors formed a separate group. Thus, instead of a general ‘Neuroticism’ component, in our case we had two independent components (‘Relaxed’ and ‘Have phobias’). This is also in line with the assumption that there is an overlap between phobic behaviors and other fear-related problems, however these do not necessarily co-occur^21^. However, it is also possible that they are only the consequence of the slightly different statistical methods.

Interestingly, in our previous study we did not find a relationship between the behavior during an outdoor separation test and the dog’s obedience^12^. However, here we found that dogs that gained higher scores in the ‘Obedient’ factor spent more time at the chair of the owner and they started to scratch the door later. On one hand it is very likely that these owners spend more time with their dogs thus they have a more balanced relationship with them, or these dogs are more trained which has a known beneficial effect on problematic behaviors^48,49^. These dogs stayed where their owner had spent his/her time before he/she left instead of standing at the door, thus they probably were less stressed. Alternatively, even though the owner did not give any command or verbal cue, the dog might have perceived the test situation as a task or exercise. However, in the outdoor separation test the dogs were tethered to a tree meanwhile their owner left them alone—this is a more stressful situation as the tree cannot be regarded as any form of ‘secure base’, unlike the owner’s chair with his/her odor on it in the indoor test.

Among the limitations of this study, we can mention that the assessment of frustration and fear-related behaviors of the subjects was done with the help of a questionnaire. Although dog owners can be considered as the closest observers of the behavior of their canine companions, they are untrained and can be often subjective source of information. At the time of our data collection there was no widely used method to describe the frustration tendencies of dogs, thus our questionnaire might not cover every aspect of it. Since then, McPeake and colleagues^23^ developed the Canine Frustration Questionnaire what might be a useful tool for further investigating of possible connection between frustration-related behaviors and separation stress. Besides, as a possible future step, along with testing dogs’ separation-related behavioral responses, one could try assessing the subjects’ reactions to such stimuli that have the potential to elicit either fear, or frustration. This would provide a more empirical approach to test the separation-related reactions in subjects that are more prone to fear or frustration.

Our results might be particularly relevant from the aspect of animal welfare, and agreeing with de Assis et al.^16^, we emphasize the importance of the recognition of the heterogeneity of this behavioral problem. Either in the case of designing empirical research, or during the development of individualized treatment strategies, one should include detailed mapping of the individual dog’s behavior and the background of it, specifically the description of the prevalence of other contexts where the dog might experience similar affective states as during separation.

## Materials and methods

### Ethical statement

Owners of the dogs were informed about the goals and circumstances of the experimental procedure a priori and they were present during the tests. We informed them that they could interrupt the experiment and reconsider their participation if—by their judgement—the test was too stressful for the dog. Their informed consent was obtained in written form via filling and signing the Department of Ethology’s standard consent form. The tests were performed in accordance with the Hungarian regulations on animal experimentation and the Guidelines for the use of animals in research described by the Association for the Study Animal Behaviour (ASAB) and ARRIVE.

All experimental protocols were approved by the Animal Welfare Committee of the Eötvös Loránd University and the National Scientific Ethical Committee on Animal Experimentation (Ref. no.: PEI/001/1056–4/2015). Informed consent was obtained from all subjects (human) for their involvement in study. Additionally informed consent was obtained from the dog owners to involve their dogs in the study.

### Human ethics

This research was approved by the National Research Ethics Committee (PE/EA/55-4/2019) and was carried out in accordance with the Declaration of Helsinki. Informed consent was obtained from all dog owners who completed our online questionnaire with an Institutional Review Board-approved protocol.

### Questionnaire

The questions (see Supplement) were taken from the already published questionnaires of Temesi et al.^45^, Bálint et al.^46^ and Lenkei et al.^12^. The questions related to the fearfulness were based on the consensus questionnaire of Temesi and colleagues who synthetized previous studies about different aspects of fear in dogs^50–54^. Besides the basic demographic information of the dog and the owner, the questionnaire contained questions about the occurrence and symptoms of SRD, and also about other possible problematic behaviors of the dog. The second section of the questionnaire contained questions about situations where the dog might act fearful (e.g.: Dog acts anxiously or fearfully when approached directly by an unfamiliar dog of smaller size) or show frustrated behavior (e.g.: If the dog wants to obtain something, it pursues persistently or even aggressively). In the case of these questions, the owner’s responses were measured on a Likert-scale from 1 (“not typical at all”/“never happens”) to 5 (“completely typical”/“happens all the time”). The questionnaire was available for the participants online and it was advertised via social media. We recorded a total of N = 397 entries to the questionnaire, from which N = 392 were used for the analysis (N = 5 entries were removed because of duplicate participation). We invited owners to the behavior test who indicated in the questionnaire that they would willingly participate in such events.

### Behavior test **(**based on Konok et al.^31^**)**

#### Subjects

We tested N = 66 subjects, however N = 10 owners did not complete the questionnaire, therefore we included only those to the analysis that had both the behavioral and questionnaire data (N = 56). The subjects were more than 1 year old family dogs (mean age in years ± SD: 6.3 ± 3, sex ratio: N = 27 males, N = 29 females; see Table 3). In the sample 13 dogs had owner reported SRD that was based on a Yes/No question in the questionnaire. The methods of the behavior tests were accepted by the Animal Welfare Committee of the Eötvös Loránd University (Ref. no.: PEI/001/1056-4/2015). The owners were informed about the aims and the methods of the experiments and that they were allowed to interrupt the tests anytime they feel the situation was too stressful for their dog.

**Table 3:** The demographic data of the subjects who participated in the separation test (N = 56).

Name	Breed	Age (years)	Sex	Reproductive status	SRD
1	Belgian Shepherd (Tervueren)	7	Male	Neutered	No
2	Mixed	6	Male	Neutered	Yes
3	Border Collie	14	Female	Neutered	No
4	Whippet	8	Male	Intact	No
5	Mixed	5	Male	Intact	No
6	Yorkshire Terrier	5	Female	Neutered	No
7	Mixed	2	Male	Neutered	No
8	Standard Schnauzer	5	Female	Intact	No
9	Mixed	5	Male	Neutered	No
10	German Pointer	5	Male	Neutered	No
11	German Shepherd Dog	5	Male	Neutered	No
12	Mixed	3	Female	Neutered	No
13	Mixed	6	Male	Neutered	No
14	Golden Retriever	2	Male	Intact	Yes
15	Belgian Shepherd (Tervueren)	4	Male	Intact	No
16	Mixed	4	Female	Neutered	No
17	Labrador Retriever	6	Male	Neutered	No
18	Mixed	7	Male	Neutered	Yes
19	Mixed	4	Female	Neutered	No
20	Azawakh	8	Male	Neutered	No
21	Mixed	8	Female	Neutered	No
22	Mixed	5	Male	Neutered	Yes
23	Mixed	11	Female	Neutered	Yes
24	Mixed	5	Female	Neutered	Yes
25	Vizsla	10	Female	Neutered	Yes
26	Mudi	2	Female	Intact	No
27	Boxer	8	Female	Neutered	No
28	Mixed	3	Female	Neutered	No
29	Bull Terrier	9	Female	Neutered	No
30	Border Collie	8	Male	Neutered	No
31	Dachshund	5	Female	Neutered	No
32	Hovawart	7	Male	Intact	No
33	Dachshund	6	Female	Intact	No
34	West Highland White Terrier	3	Female	Neutered	No
35	Great Dane	7	Female	Neutered	Yes
36	Hovawart	11	Female	Neutered	No
37	Mixed	7	Male	Neutered	No
38	Dachshund	10	Male	Neutered	No
39	Mixed	14	Male	Neutered	Yes
40	Cavalier King Charles Spaniel	7	Female	Neutered	No
41	Vizsla	9	Male	Neutered	No
42	Pumi	6	Female	Neutered	Yes
43	Mixed	5	Male	Neutered	No
44	Mixed	13	Female	Neutered	No
45	Great Dane	4	Female	Neutered	Yes
46	Whippet	2	Female	Neutered	Yes
47	Mudi	3	Female	Intact	No
48	Biewer Yorkshire Terrier	8	Female	Intact	No
49	Mixed	5	Male	Neutered	No
50	Spanish Galgo	10	Male	Neutered	Yes
51	Weimaraner	10	Male	Neutered	No
52	Golden Retriever	4	Male	Intact	No
53	Whippet	4	Male	Intact	No
54	Belgian Shepherd (Groenendael)	7	Male	Intact	No
55	Small Münsterländer	1	Female	Intact	No
56	Mixed	3	Female	Neutered	No

These dogs were all included to the analysis as their owners also completed the questionnaire about them.

#### Procedure

Dogs were tested in a room (6.27 m × 5.40 m) that was empty except for a chair. The owner entered the room with the dog on leash. After taking off the leash the dog was free to explore the room during the whole test. At first the owner sat on the chair and did not initiate any interaction with the dog. After 1 min elapsed (measured with a stopwatch by the owner), he/she left the room without any interaction with the dog, leaving the dog’s leash on the chair. The dog was alone in the room for 3 min while its behavior was recorded with a digital camera system and two microphones. After the 3 min elapsed the owner returned, greeted and petted the dog. We only used for behavior coding the interval that started when the owner closed the door after leaving the room and finished when he/she opened it again.

#### Data analysis

The behavior coding of the videos was performed by Solomon Coder (beta 17.03.22 copyright by András Péter). Table 4 shows the coded behaviors. To check the reliability of the coding method an independent observer coded 20% of randomly chosen videos. We calculated Cohens’s Kappa statistics for each behavior’s variables by taking 20% random samples. This procedure was repeated 100 times and we averaged the calculated values (Vocalization: *k* = 0.7; Distance: *k* = 0.8; Orientation: *k* = 0.664; Position: *k* = 0.881; Exploration: *k* = 0.645; Rearing: *k* = 0.835; Tail-wagging: *k* = 0.728; Scratching: *k* = 0.795). We also calculated an overall mean Kappa value (*k* = 0.76) indicating substantial agreement.

**Table 4:** The description of the coded behaviors of the separation test (we used the same coding as Marx et al.^26^ did).

Behavior label	Definition	Behavior category	Variable type
Stand	The dog is on all four feet, not moving	Position	Duration
Sit	The dog's haunches are on the ground, but the elbows are not		Duration
Lay down	The dog's elbows and sternum or side touch the ground		Duration
Move	The dog is moving, walking, or running, 2–3 paws are on the ground the whole time		Duration
Chair distance	The dog is near to the chair, within one body length	Proximity/distance	Duration
Door distance	The dog is near to the door, within one body length		Duration
Rearing up against the door/wall	The dog stands on his/her back feet and puts the forelegs on the doors or the walls	Rearing	Duration, Latency
Scratching	The dog scratches the doors or the walls, with his/her forelegs, or tries to open the door by scratching the handle	Scratching	Duration, Latency
Orientation towards the door	The dog's head is pointing towards the door	Orientation	Duration
Orientation towards the chair	The dog's head is pointing towards the chair		Duration
Exploration in general	Activity directed toward physical aspects of the environment, including sniffing, close visual inspection, distal visual inspection, and gentle oral examination such as licking	Exploration	Duration
Chair exploration	Activity directed towards the chair, including sniffing, close visual inspection, distal visual inspection and gentle oral examination such as licking		Duration
Door exploration	Activity directed towards the door, including sniffing, close visual inspection, distal visual inspection, and gentle oral examination such as licking		Duration
Tail-wagging	The dog is moving its tail constantly [not just because of the dogs’ movement (walking or running)]	Tail-wagging	Duration
Barking/yelping	A loud, short, wide pitch range sound with inverted U shaped pitch contour	Vocalization	Duration, Latency
Other vocalization	Other types of vocalizations that are not in the other categories (growling, howling, moaning, coughing, sneezing etc.)		Duration, Latency
Whining	High-pitched, relatively tonal, short and cyclic or elongated vocalizations. (Excluded from the behavior PCA)		Duration, Latency
Panting	A noise made by the dog, which sounds like a loud, moderate to rapid, open-mouth respiration		Duration

#### Statistical analysis

The statistical analyses were done in R environment^55^ using RStudio^56^. We performed two separate Principal Component Analyses (psych package principal function^57^ with oblimin rotation) on the 59 questions of the second section of the questionnaire and the separation test (based on Marx et al.^26^ full sample of the analysis: N = 167). For the questionnaire, as the questions were not continuous but Likert scales, we used polychoric correlation matrix to calculate the PCA. We ran parallel analysis (paran) in each cycle to re-determine the number of the extracted components^58^. For each factor, we calculated Cronbach’s alpha values. Based on the components we calculated behavior scores for each subject for further analysis. Later using the calculated factor scores we ran separate General Linear Models. In case of behaviors where normal distribution was found (whine_door, chair) and where boxcox based power transformation was applied (bark_door) we used General Linear Models (lm function), while in cases where the boxcox analysis suggested log transformation (esc, sit) we applied Generalized Linear Models (glm function) with Gaussian distribution and log link^59^. To analyze the latencies (the first occurrence of whining, barking, scratching the door and rearing at the door) Cox-regressions (survival package^60,61^) to compare the behavior during the separation with the results of the questionnaire. For testing the normality, we ran Shapiro–Wilk test and we used logarithmic or box-cox transformation where it was necessary. We performed backwards model selection based on *p* values. Here we report the results of the final models.

## Supplementary Information

Supplementary Tables.

## Data Availability

Data will be available upon request.
