# Supplementary material for: Separation-related behavior of dogs shows association with their reactions to everyday situations that may elicit frustration or fear
Source: Sci Rep. 2021 Sep 28;11:19207. doi: 10.1038/s41598-021-98526-3 (PMC8479053; doi:10.1038/s41598-021-98526-3)
Supplement: Supplementary file 1 — Supplementary Tables. [file 41598_2021_98526_MOESM1_ESM.docx]

Supplementary Tables

Table S1 Correlation matrix of the components resulted from the PCA analysis of the questionnaire

|  | Relaxed | Obedient | Fear Dogs | Fear humans | Demanding | Protest dislike | Have phobias |
| --- | --- | --- | --- | --- | --- | --- | --- |
| Relaxed | 1 | 0.25878317 | -0.40096614 | -0.34511896 | -0.09222946 | -0.3057281 | -0.26610161 |
| Obedient | 0.25878317 | 1 | -0.12340901 | -0.09050121 | -0.24240216 | -0.2979655 | -0.05100357 |
| Fear Dogs | -0.40096614 | -0.12340901 | 1 | 0.43414535 | 0.04649283 | 0.1689409 | 0.17810891 |
| Fear humans | -0.34511896 | -0.09050121 | 0.43414535 | 1 | 0.09497785 | 0.1518897 | 0.2290652 |
| Demanding | -0.09222946 | -0.24240216 | 0.04649283 | 0.09497785 | 1 | 0.2470724 | 0.01014597 |
| Protest dislike | -0.30572814 | -0.29796552 | 0.16894093 | 0.15188972 | 0.24707242 | 1 | 0.0972553 |
| Have phobias | -0.26610161 | -0.05100357 | 0.17810891 | 0.2290652 | 0.01014597 | 0.0972553 | 1 |

Table S2 Correlation matrix of the components resulted from the PCA analysis of the behavior test

|  | Chair | Escape | Whine - door | Bark - Wagging | Sit |
| --- | --- | --- | --- | --- | --- |
| Chair | 1.000 | 0.004 | -0.030 | -0.085 | -0.051 |
| Escape | 0.004 | 1.000 | 0.129 | -0.012 | -0.054 |
| Whine - door | -0.030 | 0.129 | 1.000 | 0.025 | -0.145 |
| Bark - Wagging | -0.085 | -0.012 | 0.025 | 1.000 | 0.058 |
| Sit | -0.051 | -0.054 | -0.145 | 0.058 | 1.000 |
